# ANATOMICAL STUDY OF THE INSERTION OF THE TRICEPS BRACHII TENDON IN THE OLECRANON THROUGH MAGNETIC RESONANCE IMAGING

**DOI:** 10.1590/1413-785220263404e293218

**Published:** 2026-07-17

**Authors:** José Renato Negrão, Arnaldo Amado Ferreira, Olavo Pires de Camargo

**Affiliations:** 1 Universidade de Sao Paulo, Faculdade de Medicina, Hospital das Clinicas (HC-FMUSP), Departamento de Ortopedia e Traumatologia, Sao Paulo, SP, Brazil.

**Keywords:** Magnetic Resonance Imaging, Tendons, Olecranon Process, Retrospective Studies, Imageamento por Ressonância Magnética, Tendões, Olécrano, Estudos Retrospectivos

## Abstract

**Introduction:**

The triceps brachii muscle (TBM) is the main muscle of the posterior aspect of the arm, occupying most of the extensor compartment. Proximally, it is composed of three heads: long, lateral, and medial, and presents a single tendon. Its insertion in the olecranon region remains controversial. Magnetic resonance imaging (MRI) assessment and detailed knowledge of this insertion may assist in orthopedic surgical reconstructions.

**Objective:**

To evaluate the anatomical aspect of the insertion of the triceps brachii tendon (TBT) into the olecranon regarding tendon insertion.

**Methods:**

*In vivo* MRI studies of patients were retrospectively evaluated. Two radiologists assessed 44 MRI scans from patients aged 20 to 50 years, with no previous triceps brachii tendon injuries or other TBT tendon pathologies.

**Results:**

There was agreement between the observers in the MRI scans evaluated, and the TBT insertion was found to be single.

**Conclusion:**

The triceps brachii tendon presented a single insertion into the olecranon. ***Level of Evidence III; Retrospective Comparative Study.***

## RESUMO

**Introdução:**

O músculo tríceps braquial (MTB) é o principal músculo da porção posterior do braço, preenchendo a maior parte do compartimento extensor. Na porção proximal, é composto por três cabeças: longa, curta e medial, apresentando um único tendão. Sua inserção na região do olécrano ainda é controversa. O estudo por ressonância magnética (RM) e o conhecimento pormenorizado dessa inserção auxiliam nas reconstruções cirúrgicas ortopédicas.

**Objetivo:**

Avaliar o aspecto anatômico da inserção do tendão do tríceps braquial (TTB) no olécrano em relação à inserção tendínea.

**Método:**

Estudos de RM de pacientes in vivo, avaliados de forma retrospectiva. Dois radiologistas avaliaram 44 exames de RM de pacientes com idade entre 20 e 50 anos, sem lesões prévias do tendão do tríceps braquial ou outras patologias tendíneas do TTB.

**Resultados:**

Houve uma concordância entre os observadores nas RM avaliadas e a inserção do TTB apresentou-se ser único.

**Conclusão:**

O tendão do tríceps braquial apresentou uma única inserção no olécrano. ***Nível de Evidência III; estudo Retrospectivo Comparativo.***

## INTRODUCTION

The triceps brachii muscle (TBM) is the main muscle in the posterior portion of the arm, filling most of the extensor compartment. The proximal portion is made up of three heads: long, short and medial, thus giving rise to its name.^1–2^ Detailed knowledge of this insertion helps in orthopedic reconstruction, since the existing orthopedic literature is still very controversial.^1–3^

The triceps brachii muscle has three distinct muscle bellies and tendons. The current characteristics only present the description of a tendon inserting into the olecranon. The origin of the long head of the triceps is a flattened tendon of the infraglenoidal tubercle of the scapula, fusing above with the joint capsule of the shoulder. Its muscle fibers descend medially to the short head and superficially to the medial head, joining them to form a common tendon. ^1–3^

The lateral head of the triceps arises from a flattened tendon arising from a narrow, linear, oblique ridge on the posterior surface of the shaft of the humerus and the lateral intermuscular septum, converges to the common tendon. After, the medial head is superimposed posteriorly by the short and long heads, exhibiting an extensive origin on the posterior surface of the humeral shaft below the radial groove, in the insertion plane of the teres major muscle, which is up to approximately 2.5 cm from the trochlea. Some muscle fibers reach the olecranon directly; others converge on the common tendon.^3^

The insertion tendon of the triceps brachii (TBT) begins in the medial portion of the muscle, having two blades: one superficial and the other deep. After the convergence of the muscle fibers, these two blades come together above the elbow and most fibers will insert into the superior surface of the olecranon.^3^

The study of TTB insertion in cadavers made a correlation between the anatomical dissection with the histological study, reporting that there were two insertions during dissection but only one during histological evaluation.^14–6^

Magnetic resonance imaging (MRI) is growing in clinical practice and being the key method for study the TBT in the olecranon because is a non-invasive modality and has high resolution between tissues. It is mainly used in cases of total or partial tendon ruptures. The objective of the study is to demonstrate in-vivo patients, using MRI, how the distal insertion of the TBT into the olecranon effectively occurs with the purpose of assist the orthopedist in tendon reconstruction.

## MATERIALS AND METHODS

The study was carried out at the Institute of Orthopedics and Traumatology of the Hospital das Clínicas of the Faculty of Medicine of the University of São Paulo - FMUSP. In this study, 105 MRI exams of routine patients at the Institute of Orthopedics and the "Hospital das Clínicas of FMUSP were randomly analyzed. 61 exams were excluded from the study, due to the exclusion criteria used, such as age, that is, 44 exams. Factors such as age between 20 and 50 years and other clinical and surgical changes found in the studies meant that the number of exams was necessarily changed. This elbow study is a retrospective analysis where they were carried out using 1.5 Tesla HDX® equipment (GE Healthcare, Waukesha, WI, USA) with a 1.5 Tesla HD Knee Transducer Coil (Channel TR Knee). The MRI protocol of the Radiology and Imaging Diagnostic Service of the Institute of Orthopedics and Traumatology adopted to study the insertion of the triceps brachii will be described below: a) Sagittal, axial and coronal planes; b) Fast spin echo technique; with T1 and T2 weighted images (matrix: 256, number of excitations (nex): 3, field of view (FOV) of 8 cm, thickness of 3.0 mm and gap of 0.5 mm). The images acquired during the exams were inserted into a data capture system (Pictures Archive Computer System Station – PACS). The positioning of patients, when performing the MRI examination, patients were positioned in the supine position with the arm above the head region.

All images were analyzed by two observers, radiologists with over 6 years of experience in imaging the musculoskeletal system. These evaluations were carried out independently and without prior knowledge of the results. The three sets of findings (two observers) will be used in data analysis and calculations of intra and inter observer correlations.

The images evaluated were used in the sagittal and axial planes in T1 and T2 weighted sequences. The images evaluated followed the plans produced by the Institute of Orthopedics and the "Hospital das Clínicas of FMUSP". These plans were used because they are the best sequences in relation to the information provided containing a more detailed analysis of the insertion of the triceps brachii tendon (TBT).

## RESULTS

**Figure 1:**
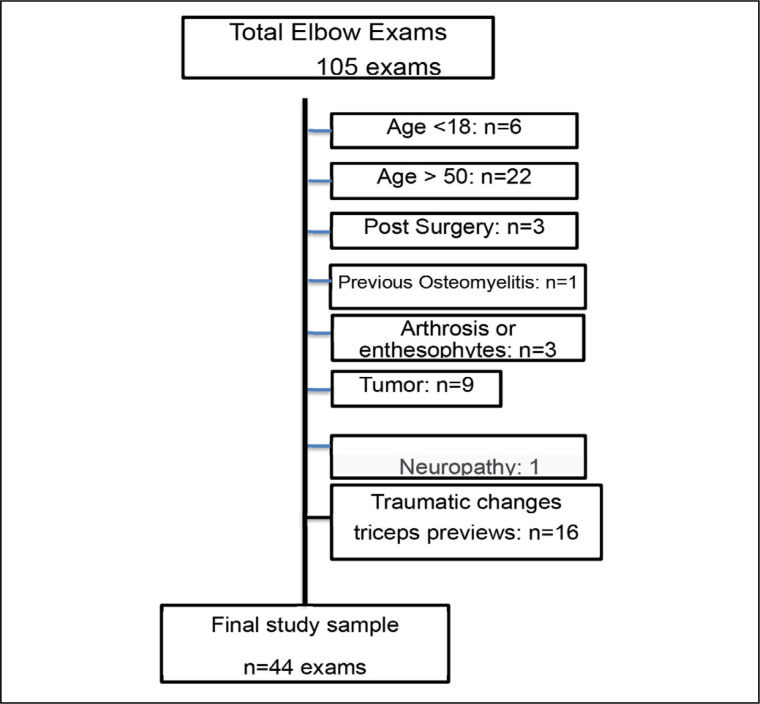
Study flowchart illustrating the exclusion criteria.

MRI elbow exams from 2012 to 2014 were eligible for the study. Therefore, from the 105 exams that were retrospectively included and after applying the study exclusion criteria, only a total of 44 exams were included, as shown in Figure 1.

According to Table 1, it shows that the exams evaluated came from MRI exams of patients aged between 20 and 50, half being male subjects. Still according to Table 1, it also shows that the age group between 41 and 50 had a higher frequency of imaging exams of the right elbow.

**Table 1:** Description of the demographic and clinical data of the 44 participating exams that made up the sample.

Age	Number (%)
Average ± standard deviation	37.9 ± 9.5
Minimum – maximum	20 – 50
**Age Group**	
20 to 30	11 (25.0)
31 to 40	13 (29.6)
41 to 50	20 (45.4)
**Gender**	
Male	22 (50.0)
Female	22 (50.0)
**MRI**	
Right Elbow	25 (56.8)
Left Elbow	19 (43.2)

Each of these 44 exams was evaluated by two independent observers who classified the insertion of the triceps brachii tendon. Table 2 presents the results of these classifications where it shows that there was classification agreement between the two observers in 42 exams (95.4%). The Kappa agreement coefficient was calculated presenting a value equal to 0.847 (95% CI: 0.641; 1.000) and can be interpreted as a very good agreement.

**Table 2:** Distribution of the 44 classifications carried out by two independent observers regarding the insertion of the triceps brachii tendon.

Classification of tendon insertion		Number (%)
Observer 1	Observer 2	
Single insertion	Single insertion	35 (79.5)
Variation	Variation	7 (15.9)
Single insertion	Variation	1 (2.3)
Variation	Single insertion	1 (2.3)

Table 3 presents the results of the classification of tendon insertion by the two independent observers according to age group. It shows that, for elbows from MRI exams aged between 20 and 30, the two observers agreed that the single insertion was in all exams. For the age group between 31 and 40, there was agreement between the two observers in 12 exams (92.3%), while for the age group between 41 and 50, they agreed on 19 MRI exams (95%). The Kappa coefficient of agreement for the age group between 31 and 40, was equal to 0.755 (95%CI: 0.307 to 1.000) and for the age group between 41 and 50, the value obtained was equal to 0.875 (95%CI: 0.638; 1,000). These coefficients can be interpreted as showing good and very good agreement, respectively.

**Table 3:** Distribution of the 44 classifications for triceps brachii tendon insertion carried out by two independent observers according to age group.

	Insertion Classification of the tendon insertion		Number(%)
Age Group	Observer 1	Observer 2	
20 to 30	Single Insertion	Single Insertion	11 (100.0)
31 a 40	Single Insertion	Single Insertion	10 (76.9)
	Variation	Variation	2 (15.4)
	Variation	Single Insertion	1 (7.7)
41 a 50	Single Insertion	Single Insertion	14 (70.0)
	Variation	Variation	5 (25.0)
	Single Insertion	Variation	1 (5.0)

According to Table 4, it shows that the agreement occurred in 20 MRI female elbows exams (90.9%) and 22 MRI male elbows exams (100%). The Kappa agreement coefficient for females was equal to 0.771 (95% CI: 0.470 to 1.000), which can be interpreted as a good agreement. For males, it can be said that the agreement between the two observers was very good.

**Table 4:** Distribution of the 44 classifications for triceps brachii tendon insertion carried out by two independent observers according to gender.

	Tendon Insertion Classification		Number (%)
Gender	Observer 1	Observer 2	
Female	Single Insertion	Single insertion	15 (68.2)
	Variation	Variation	5 (22.6)
	Single Insertion	Variation	1 (4.6)
	Variation	Single Insertion	1 (4.6)
Male	Single Insertion	Single Insertion	20 (90.9)
	Variation	Variation	2 (9.1)

According to Table 5, it shows that the agreement occurred in 23 exams (92%) for the right-side elbows and in 19 exams (100%) for the left side. The Kappa agreement coefficient for the right side was equal to 0.702 (95% CI: 0.316 to 1.000), which can be interpreted as a good agreement. For the left side, the agreement between the two observers was very good.

**Table 5:** Distribution of the 44 classifications for insertion of the triceps brachii tendon carried out by two independent observers according to the side of the elbow.

	Tendon Insertion Classification		Number (%)
Elbow Side	Observer 1	Observer 2	
Right	Single Insertion	Single Insertion	20 (80.0)
	Variation	Variation	3 (12.0)
	Single Insertion	Variation	1 (4.0)
	Variation	Single Insertion	1 (4.0)
Left	Single Insertion	Single Insertion	15 (79.0)
	Variation	Variation	4 (21.0)

### Statistical Analysis

A descriptive analysis was performed where categorical variables were summarized by the number (*n*) and percentage (%) and non-categorical variables as an average ± standard deviation, minimum and maximum values.

Agreement between the two observers was assessed by calculating the observed agreement (proportion of exams with the same classification by both observers) and the Kappa-Cohen agreement coefficient (κ) and its respective 95% confidence interval (95%CI). The Kappa coefficient varies between 0 and 1, where higher values indicate greater agreement between observers. The degree of Kappa agreement was interpreted as poor (Kappa ≤ 0.20), fair (Kappa between 0.21 and 0.40), moderate (Kappa between 0.41 and 0.60), good (Kappa between 0.61 and 0.80) and very good for Kappa values between 0.81 and 1.00 (Altman, 1991).

Statistical analysis was done using Stata/MP 18.0 (Stata-Corp, 2023. College Station, TX: Stata Corp LLC).

Altman DG (1991) Practical statistics for medical research. London: Chapman and Hall.

## DISCUSSION

**Figure 2:**
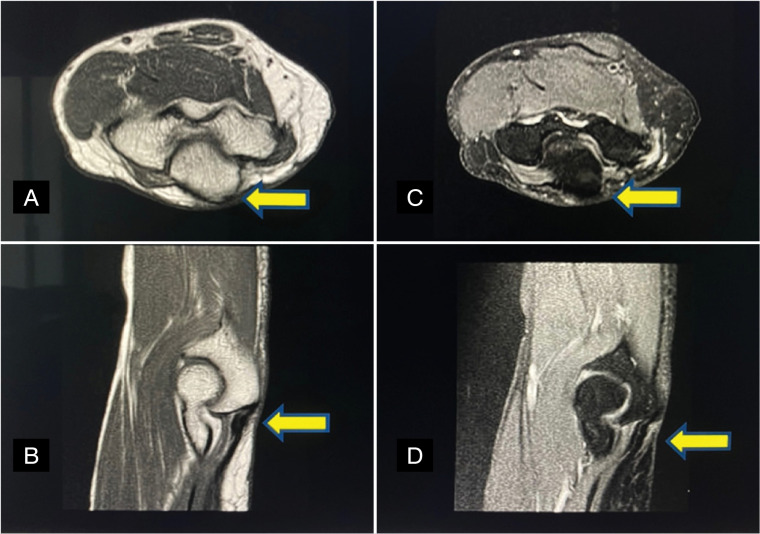
(A) and (B) T1 Sequence of axial and sagittal MRI scans show the distal triceps tendon insertion in the olecranon (yellow arrows); (C) and (D) T2 Sequence of axial and sagittal show the distal triceps insertion in the olecranon (yellow arrows).

In the literature that was evaluated, several studies showed findings of the triceps brachii in cadavers together with dissections and histological studies. However, this is the first study that evaluates the anatomical findings of the triceps brachii retrospectively from in-vivo examinations, according to Figure 2.

The MRI study of the insertion of the triceps brachii tendon has been the key topic of several studies. The main objective of this study is to assist orthopedists and radiologists in the surgical correction of the triceps brachii tendon and to expand the analysis of its insertion. This finding should be considered in clinical practice to achieve greater assurance and more positive results in reconstruction surgery for triceps brachii tendon injuries.

This study was carried out using MRI as this method presents better resolution between tissues and does not use ionizing radiation. The radiography (x-ray) study does not present details of soft tissues that are necessary to enable the evaluation of the tendon. Computed tomography study would not be able to participate in this study because it also does not present fine necessary details and both methods use ionizing radiation. The ultrasound study presents tissue details without using ionizing radiation but, it is an examiner-dependent method and has limitations such as the low reproducibility of the methodology. ^8–12^

Traditional anatomical descriptions indicate three heads of the triceps brachii muscle called the long head, the lateral head, and the medial head, which insert into the olecranon. The long head originates basically from the infraglenoidal tubercle of the scapula and inferior glenoumeral joint capsule. The lateral head has three points of origin: the posterior surface of the humerus between the insertion of the teres minor tendon and the superior surface of the spiral groove, the lateral edge of the humerus, and the lateral intermuscular septum. The head originates from the posterior surface of the humerus, distal to the spiral groove and medial aspect of the intermuscular septum. The medial and lateral heads serve only as extensors of the elbow, while the long head assists in adduction and extension of the glenohumeral joint.^12–15^

The triceps brachii tendon has a different characteristic from other tendons, as it originates from three muscle bellies. These muscle bellies originate from specific bone locations as described previously and are inserted into a single location, that is, the olecranon. Therefore, in MRI, this characteristic takes on a bipartite appearance.^13–15^

An accurate understanding of the distal anatomy of the triceps tendon insertion is clinically important in surgical planning for procedures such as reduction of displaced fractures of the distal portion of the posterior surface of the humerus, olecranon osteotomies and repairs of partially or completely torn triceps tendons. Furthermore, it is important to know that the most common traumatic injuries associated with triceps tendon, is a fracture of the radial head, probably due to the same injury mechanism (fall with the arm extended). Other possible associated injuries are medial collateral ligament tears or laxity, compression of the ulnar or radial nerve as well as fractures of the proximal humerus. Immediate surgical repair of an acute tear (less than six weeks) is generally recommended with reinsertion of the triceps tendon using suture anchors. When treatment is delayed, the degenerated and fragile appearance of the stump can prevent reinsertion of the tendon. ^14–16^

In this study, a detailed assessment of the triceps brachii tendon was carried out retrospectively in in-vivo MRI studies, resulting in an assertive assessment in relation to some studies that used cadavers to perform elbow studies.^13^

**Figure 3:**
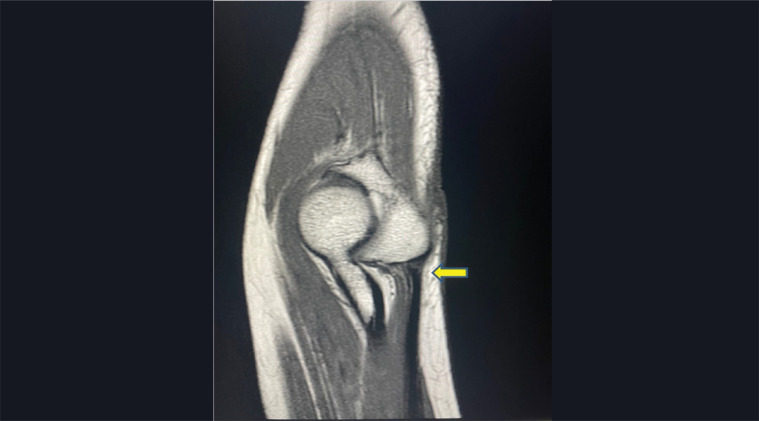
T1 Sequence MRI sagittal scan showing of the distal triceps insertion in the olecranon (yellow arrow).

**Figure 4:**
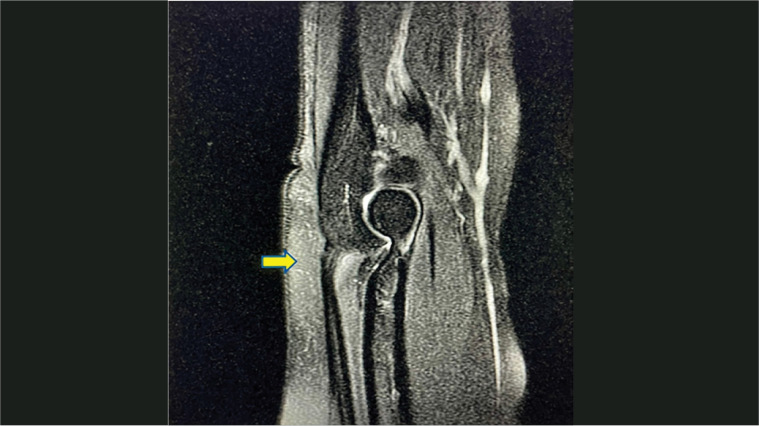
T2 Sequence MRI sagittal scan showing the distal triceps tendon insertion in the olecranon (yellow arrow).

It is important to remember that the main difference of this study in relation to others already carried out using MRI is that this study was evaluated by examiners as well as evaluation of retrospective in vivo exams, showing a single insertion of the triceps brachii in Figures 3 and 4. Therefore, there are no changes in relation to the post-mortem. For example, with a reduction in the level of hydration of the tendon and cadaver stiffness as well as possible previous degenerative changes, these changes that were excluded from the study, could compromise the evaluation.

Although Belentani et al.,^13^ using a different MRI protocol, describes the distal insertion of the triceps tendon as a bipartite appearance. However, histological analysis in this study provided definitive evidence of a single distal insertion.^13^ This same observation is also reported by Hayter and Adler.^6–8^ t was identified that there is a tendon of the distal medial head that joins the lateral and long tendons (sets). In the future with better and enhanced MRI equipment, this tendon could be more visible since, currently; it is too small to authenticate its existence. However, even with this aspect the insertion was identified as unique.

In MRI studies the main tendon of the medial head was not identified. Barco et all^15^ indicates that the medial head tendon is inconsistent or too small to be measured. Another possible explanation for the different MRI and histology results analyzed in this study^14^ is the histological sections. They showed the tip of the insertion where the central tendon is very close to the medial head tendon and, probably, there could be fusion of the fibers of the three heads. The rolled edge represents a deep medial thickening of the central tendon that receives contributions from the medial head.^12^ However, a survey of the integrity of all insertion sites of the medial head also identifies a single insertion in the olecranon in the deep muscle component.^14–17,18^

A distal pre-tricipital space found in anatomical dissections, probably a split, demonstrates a bipartite appearance, as previously described in the literature.^11^ As in the study by Akamatsu et. all,^19^ some MRI scans spotted the existence of the pre-tricipital space. However, it is also not clearly identified by observing radiologists.^19^

According to a study by Kholinne et. all,^21^ this current study also has limitations. However, this retrospective study, with a greater number of patients in vivo, provides a greater range of analysis because the MRI studies were carried out on living patients and not associated with cadavers. Therefore, the evaluation is not hampered by reduced post-mortem hydration, as well as cadaver stiffness, which we believe may present less accurate results.^20–21^

The area is not measured in the MRI study because the traditional measurement at tendon insertion is calculated by the greatest length x width. Thus, one can accurately underestimate the 3D structure of its actual insertion area.

The lack of histological evaluation or dissection is due to the impossibility of performing histological studies or dissection on living patients. Despite the previously mentioned limitations, this study provided us with a clear view that there is a single insertion of the triceps brachii tendons into the olecranon, even taking into consideration that the tendons have a different form of insertion.

Therefore, this information provided in this study can help to clarify the diagnosis of partial ruptures of the distal triceps brachii tendon, helping to provide a reliable satisfactory result for the patient.

## CONCLUSION

The detailed study of the insertion region of the triceps brachii tendon using MRI identifies and confirms that the triceps brachii tendons have a single insertion into the olecranon.

## Data Availability

The underlying contents of the research text are contained in the manuscript.

## References

[1] Testut L, Latarjet A (1979). Tratado de Anatomia Humana.

[2] Gray H (1995). Gray's Anatomy. The Anatomical Basis of Medicine and Surgery.

[3] Gray H (2008). Gray's Anatomy.

[4] Sampath SC, Sampath SC, Bredella MA (2012). Magnetic resonance imaging of the elbow: A Structured Approach. Sports Health.

[5] Bucknor M, Stevens K, Steinbach L (2016). Elbow imaging in sport: Sports imaging series. Radiology.

[6] Athwal GS, McGill RJ, Rispoli DM (2009). Isolated avulsion of the medial head of the triceps tendon: an anatomic study and arthroscopic repair in 2 cases. Arthroscopy.

[7] Chan APH, Lo CK, Lam HY, Fung KY (2009). Unusual traumatic triceps tendon rupture: a word of caution. Hong Kong Med J.

[8] Hayter CL, Adler RS (2012). Injuries of the elbow and the current treatment of tendon disease. AJR Am J Roentgenol.

[9] Sharma SC, Singh R, Goel T, Singh H (2005). Missed diagnosis of the triceps tendon rupture: a case report and review of literature. J Orthop Surg.

[10] Levy M (1987). Repair of triceps tendon avulsions or ruptures. J Bone Joint Surg Br.

[11] Keener JD, Chafik D, Kim HM (2010). Insertional anatomy of the triceps brachii tendon. J Shoulder Elbow Surg.

[12] Madsen M, Marx R, Millett P, Rodeo SA, Sperling JW, Warren RF (2006). Surgical anatomy of the triceps brachii tendon anatomical study and clinical correlation. Am J Sports Med.

[13] Belentani C, Pastore D, Wangwinyuvirat M, Dirim B, Trudell DJ, Haghighi P (2009). Triceps brachii tendon: anatomic-MR imaging study in cadavers with histologic correlation. Skeletal Radiol.

[14] Negrão JR, Mogami R, Ruiz FAR, Wagner FV, Haghighi P, Ward SR (2020). Distal insertions anatomy of the triceps brachii muscle: mri assessment in cadaver specimens employing histologic correlation and play-doh® models of the anatomic findings. Skeletal Radiol.

[15] Morrey BF (1993). Anatomy of the elbow joint and its disorders.

[16] Jacobson JA, Lebson PJL, Jeffers AW, Fessel D, Hayes C (2001). Ulnar nerve dislocation snapping triceps syndrome: diagnosis with dynamic sonography-report of three cases. Radiology.

[17] Evans RC (2003). Exame físico ortopédico ilustrado.

[18] Keener JD, Chafik D, Kim M, Galatz LM, Yamaguchi K (2010). Insertional anatomy of the triceps brachii tendon. J Shoulder Elbow Surg.

[19] Akamatsu FE, Negrão JR, Rodrigues MB, Itezerote AM, Saleh SO, Hojaij F (2020). Is there something new regarding tríceps brachii muscle insertion?. Acta Cir Bras.

[20] Celli A (2015). Triceps tendon rupture: knowledge acquired from the anatomy to the surgical repair. Musculoskelet Surg.

[21] Kholinne E, Kwak JM, Heo Y, Hwang SJ (2022). The anatomic – magnetic resonance imaging study of distal triceps brachii tendon. J Orthop Surg (Hong Kong).

